# Incorporating virtual fencing to manage yearling steers on extensive rangelands: spatial behavior, growth performance, and enteric methane emissions

**DOI:** 10.3389/fvets.2025.1637190

**Published:** 2025-09-10

**Authors:** Edward J. Raynor, Anna M. Shadbolt, Melissa K. Johnston, David J. Augustine, Justin D. Derner, Sean P. Kearney, John P. Ritten, Nathan D. Delay, Pedro H. V. Carvalho, Juan de J. Vargas, Sara E. Place, Kim R. Stackhouse-Lawson

**Affiliations:** ^1^Department of Animal Sciences, Colorado State University, Fort Collins, CO, United States; ^2^Rangeland Resources and Systems Research Unit, US Department of Agriculture−Agricultural Research Service, Fort Collins, CO, United States; ^3^Rangeland Resources and Systems Research Unit, US Department of Agriculture−Agricultural Research Service, Cheyenne, WY, United States; ^4^Thunder Basin Grasslands Prairie Ecosystem Association, Douglas, WY, United States; ^5^Department of Agricultural and Resource Economics, Colorado State University, Fort Collins, CO, United States

**Keywords:** animal distribution, data fusion, GPS and AHCS, rangeland enteric emissions, shortgrass steppe, spatial distribution management, virtual fencing technology and GreenFeed

## Abstract

We examined the spatial movement behavior, growth rates, and enteric CH_4_ emissions of yearling beef cattle in response to spatial distribution management with virtual fencing (VF) in extensive shortgrass steppe pastures. Over the 110-d grazing season (mid-May to early September), 120 British-breed stocker steers (~12 months of age; mean body weight [BW] 382 kg ± 35) were grazed with VF management (active VF collars) or free-range (non-active VF collars) in two pairs of ~130 ha physically fenced rangeland pastures (i.e., VF-managed vs. control). One pair was associated with a diverse mosaic of soil types supporting alkalai sacaton (*Sporobolus airoides* [Torr.] Torr.), blue grama (*Bouteloua gracilis* [Willd. Ex Kunth] Lag. Ex Griffiths), and needle-and-thread (*Hesperostipa comata* [Trin. &Rupr.] Barkworth), while the other pasture-pair was associated with the Sandy Plains ecological site, primarily hosting western wheatgrass (*Pascopyrum smithii* [Rydb.] Á. Löve), needle-and-thread, and blue grama. Within each pair of pastures, one herd was rotated among sub-pastures using the VF system, which focused grazing on varying native plant communities over the growing season. In control pastures, steers had access to the entire pasture for the grazing season. Spatial distribution management with VF maintained steers within desired grazing areas occurred 94–99% of the time, even though five of the 60 VF-managed steers consistently made short daily excursions outside the VF boundary. In all four pastures, an automated head-chamber system (AHCS, i.e., GreenFeed) measured the enteric CH_4_ emissions of individual steers. Steers that met the criteria of a minimum of 15 AHCS visits in each of at least two VF rotation intervals were analyzed for spatial behavior, growth performance, and enteric CH_4_ emissions. Screening based on AHCS visitation requirements resulted in 15 steers (nine VF, six control) in the diverse mosaic pasture pair, and 39 (17 VF, 22 control) in the Sandy Plains pasture pair. VF management significantly reduced growth rates for all steers across both pasture pairs by an average of 9%, resulting in steers that were 7.3 kg lighter than unmanaged steers at the end of the grazing season. VF management effects on enteric CH_4_ emissions varied among rotation intervals and pasture type. In the diverse mosaic pair, VF management significantly reduced CH_4_ emissions during the first rotation interval, when VF steers were concentrated on the C_3_ grass-dominated plant community, but increased emissions in the second and third intervals when VF steers were concentrated on C_4_ grass-dominated areas. In the Sandy Plains pasture pair, where cattle were rotated between sub-pastures with and without palatable four-wing saltbush (*Atriplex canescens* [Pursh] Nutt.) shrubs, VF management reduced CH_4_ emissions in three of four rotations as well as over the full grazing season. CH_4_ emissions intensity increased with VF management in the diverse mosaic, but not in the Sandy Plains pastures. Overall, our findings show VF management (1) controlled animals spatially within sub-pastures, (2) did not improve growth performance but rather decreased it, (3) did not consistently reduce enteric CH_4_ emissions, and (4) tended to increase emissions per kg of product via lowering steer growth performance. While some have posited that VF is a potential tool to reduce enteric emissions, our findings suggest VF management is not a straightforward solution for mediating the relationships between forage resources, growth performance, and enteric CH_4_ emissions of stocker steers on extensive rangeland. Furthermore, our fusion of animal GPS tracking, growth rates and AHCS data indicated that differences in spatial behavior and weight gain were consistent between VF-managed and control steers irrespective of their AHCS-acclimation status, supporting the perspective that AHCS-based gas flux measurements are a valid means of estimating enteric emissions in extensive rangelands.

## 1 Introduction

Spatial behavior of livestock in extensive systems lies at the nexus of space use, foraging decisions, animal growth performance, and eventual enteric CH_4_ emissions (1). Management of the spatial distribution of cattle through physical or electric fencing is one of the most widespread practices in extensive rangelands, and the influence such management has on stock density and forage allocation is well known to influence animal growth performance (2–4). Over the last two decades, there has been a rapid growth in the development of precision livestock farming technologies, including on-animal or wearable sensors for grazing livestock. Most wearable sensor systems are passive, providing data to livestock managers that includes diagnostics for health, nutritional, or reproductive states or events, such as estrus and calving (5, 6). Animal location is often a crucial element of grazing systems, with data from Geographical Navigation Satellite Systems (GNSS, frequently referred to as GPS [Global Positioning System]) allowing the creation of map-based visualizations (7).

Virtual fencing (VF) systems represent a significant advancement over passive sensors, offering active livestock management and precision livestock farming technology. VF systems utilize two-way digital communication between livestock managers and each animal via its wearable device, referred to as a VF collar. Managers can then actively, but remotely, manage desired grazing areas for the herd, opening or restricting movement in near real-time.

Experimentally-informed knowledge on the behavior and welfare of beef cattle under VF-based management in extensive rangeland systems is needed, as this precision livestock management technology is experiencing rapid commercial uptake (8, 9). Yet, most evaluations of VF efficacy and effects on animal behavior or performance pertain to short durations in small, intensively managed systems over ≤60-d periods [e.g., 0.2–10 ha in size; (10–12)] or to more extensive grazing systems over ≤40-d periods [e.g., 2.1–414 ha; (9, 13)].

Using VF-based management in extensive grazing systems, such as semiarid rangelands, potentially enables managers to target higher forage quality, thereby enhancing the likelihood of achieving production goals (14–16). With conventional boundary systems (i.e., physically fenced pastures) on large rangeland parcels, it is often impractical to focus grazing, even if it is deemed useful for both farming and nature conservation objectives (17). Recent literature suggests that VF can overcome many of these issues in extensive systems, where opportunities exist to easily and flexibly manage grazing at a finer spatial scale, keeping livestock well away from risk areas (which may be seasonal or temporary) using virtual exclosures or focusing herds onto specified grazing areas (17). For instance, an initial study that deployed VF collars on beef cattle to curb utilization of burned sagebrush steppe demonstrated the efficacy of this technology in modulating post-wildfire herd distribution for land restoration purposes (13). Yet, little is known about how the use of this emerging technology on extensive rangeland can affect spatial behavior, growth performance, and enteric CH_4_ emissions, the latter of which is one of the most challenging agricultural products to manage but also with the most potential to reduce the overall carbon footprint of the beef sector (18).

At the same time, prior studies of rotational grazing management in extensive systems via physical fences show that increasing stock density (number of animals per unit area at any given point in time) alters foraging behavior and reduces diet quality, which results in reduced weight gain (4, 19). Use of virtual fencing to subdivide pastures and rotate cattle sequentially through the subdivisions will increase stock density, with the magnitude of the increase depending on the size of subdivision areas. It remains unclear whether this effect of stock density on weight gain can be offset by implementing VF rotations in a manner that targets different plant communities when they are most palatable. In standard extensive grazing systems on western US rangelands, the current high costs of VF also necessitate clear economic benefits from combinations of hardware (e.g., wire fences) and labor savings or production benefits (20, 21). Yet, evidence for production benefits on extensive rangeland remains limited.

Researchers have also theorized that incorporating VF management could reduce enteric CH_4_ emissions by enhancing animal growth performance without detrimental effects on welfare (15). As reviewed by Vargas, Ungerfeld (22), research has shown that ruminants on diets with higher fiber concentrations result in greater enteric CH_4_ production (g/d) due to more favorable conditions for rumen methanogenesis. Thus, by coupling knowledge of how different forage types affect enteric emissions with grazing plans (23, 24), managers could potentially reduce enteric CH_4_ emissions of beef cattle grazing extensive rangelands through adopting VF technology.

Assessing enteric methane (CH_4_) production in extensive beef cattle production systems is one of the most challenging aspects of developing innovative, emissions-reducing production practices across the beef sector (18, 25). The cow-calf and stocker stages, where growing cattle graze forage before finishing in confinement, have been identified as the portion of the beef cattle life cycle when 89% of enteric CH_4_ emissions are produced (26). Compared to confined settings, this production stage is the most difficult for measuring and managing CH_4_ emissions (14, 27). Because enteric CH_4_ is produced during the anaerobic fermentation of organic matter in the rumen and represents an energy loss for the animal that varies between 2 and 12% of the gross energy intake (28, 29), spatial management practices that influence animal distribution and access to forage of varying quality have the potential to impact production efficiency in extensive grazing systems.

In confined settings, the ability to manage intake and monitor growth performance in near real-time has allowed managers and researchers to develop alternative diets and interventions that lower CH_4_ synthesis of fed-animal production systems (18, 27, 30) and the overall footprint of the beef cattle supply chain (29, 31, 32). Yet, the capacity to manage foraging decisions, spatial behavior, and concomitant growth performance in grazing systems is limited mainly to small, pasture-based, intensively managed grazing systems, where producers primarily manage consumption of the most digestible, nutrient-dense plant parts through physical or electric fencing (33, 34). For example, tannin-containing birdsfoot trefoil (*Lotus corniculatus* L., variety Langille) and small burnet (*Sanguisorba minor* Scop., variety Delar) have been shown to reduce enteric CH_4_ emissions and urinary nitrogen (N) excretion in intensive grazing systems (34). Additionally, targeted grazing of timothy (*Phleum* L.) or Italian ryegrass (*Lolium multiflorum* L.) at an early vegetative state has been shown to be a successful method for reducing enteric CH_4_ emissions in intensive grazing systems (35, 36). In contrast, practices for reducing the emissions of free-ranging livestock in extensive production environments remain limited (22, 27, 37–39), which is unfortunate as 78% (166 M ha) of the 214 M ha of US grazing land are extensive production systems (40). Furthermore, a recent review of feeding strategies to mediate enteric emissions in grassland systems by Vargas, Ungerfeld (22) found targeted-grazing research evaluating enteric CH_4_ emissions in extensive grazing systems was absent from the literature. Thus, there is a growing need to identify where and which precision livestock management technologies can be used to increase production and reduce emissions, partly because enteric CH_4_ emissions vary spatially and by production context (18, 26, 32).

Unlike controlled production settings, such as intensive-pasture management and confined animal feeding operations, measurements of enteric CH_4_ emissions on extensive grazing systems have been relatively difficult to obtain. Before the availability of automated head-chamber systems (AHCS, i.e., GreenFeed, C-Lock, Inc., Rapid City, SD), techniques including the sulfur hexafluoride (SF_6_) marker dilution method had been the primary means of collecting enteric emissions data on pasture (36, 41). At the same time, whole animal respiration chambers and head chambers served as the gold standard across research settings (42, 43). These tools enabled enteric CH_4_ emissions measurements, yet their ability to collect gas flux data that reflects working production systems was limited in comparison to AHCS units (41), which are deployed in both confined and open grazing environments for long-duration measurement periods [i.e., weeks to months; (44, 45)]. As opposed to previous methods, which collect animal gas flux for single-day to three-day increments without the need for handling animals, AHCS measurement periods span as long as electrical power persists and bait remains available to attract individual animals to the automated head-chamber.

The same less-invasive element that allows AHCS technology to reflect working production operations, voluntary intake of bait by unconstrained livestock, also represents a limitation, especially in grazing systems. By having free choice to graze forage and consume AHCS bait on pasture, cattle visit AHCS units at a much lower daily rate in grazing systems [e.g., 3–4.5 visits/d; (45, 46)] than observed in confined settings [(44, 45), e.g., 3–4.5 visits/d; (46), e.g., 1–2 visits/d; (47, 48)]. Moreover, the percentage of individual cattle in a herd that will routinely use the AHCS varies widely, irrespective of production setting. For example, Alemu, Shreck (45) reported 48–84% of cattle acclimated to AHCS in confinement, and acclimation rates were equally variable for intensive-managed [e.g., 34–70%; (44, 47–49)] and extensive-managed grazing systems [e.g., 46–62%; (50, 51)]. To date, the variability in routine use of AHCS units across production systems has remained a methodological issue for researchers without a firm understanding of pertinent AHCS use drivers (41, 44). However, recent efforts have identified overall behavior, including feeding behavior and between-animal variation, as key animal-oriented factors driving this variability in grazing systems (25). Nonetheless, AHCS technology remains a flexible method to collect enteric gas flux data across ruminant livestock production settings.

Here, we investigate the influence of VF on spatial behavior, growth performance, and enteric CH_4_ emissions of growing steers in an extensive livestock production system. We first evaluate the efficacy of the VF system in manipulating cattle distribution into desired sub-pastures. We then examine consequences for cattle movements on a daily basis within each rotation interval, for CH_4_ emissions at the level of rotation intervals and the full grazing season, and finally for steer weight gain and CH_4_ emissions intensity (g CH_4_ emitted per kg beef produced) over the full grazing season.

## 2 Materials and methods

### 2.1 Site description

This research was conducted at the United States Department of Agriculture – Agricultural Research Service Central Plains Experimental Range (CPER) near Nunn, Colorado (40.833333, −104.716667, 1,600 m above sea level). The site is a native shortgrass steppe comprised of cool-season (C_3_) grasses and forbs, as well as warm-season (C_4_) grasses. The dominant cool-season grasses include western wheatgrass [*Pascopyrum smithii* (Rydb.) Á. Löve] and needle-and-thread grass [*Hesperostipa comata* (Trin. & Rupr.) Barkworth]. The dominant warm-season grass is blue grama [*Bouteloua gracilis* (Willd. Ex Kunth) Lag. Ex Griffiths]. The major forb component is scarlet globemallow [*Sphaeralcea coccinea* (Nutt.) Rydb.]. The average annual precipitation is 340 mm with an average growing season of 120 d. Mean annual temperature ranges from an average low of −11.3°C in January to an average high of 31.7°C in July (52). Precipitation totals in 2024 for April to August were 78% (172 mm) of the historic growing season average [222 mm; (53)] while forage production was 61% of long-term average. The topography consists of slightly undulating plains. Soils consist of deep, well drained, fine sandy loams to loamy sands on alluvial flats and upland plains (54). All research followed the Institutional Animal Care and Use Committee protocol (#CPER-9) approved March 2024 by the USDA–Agricultural Research Service in Fort Collins, CO, USA.

### 2.2 Forage quantity and diet quality evaluation

**Figure 1:**
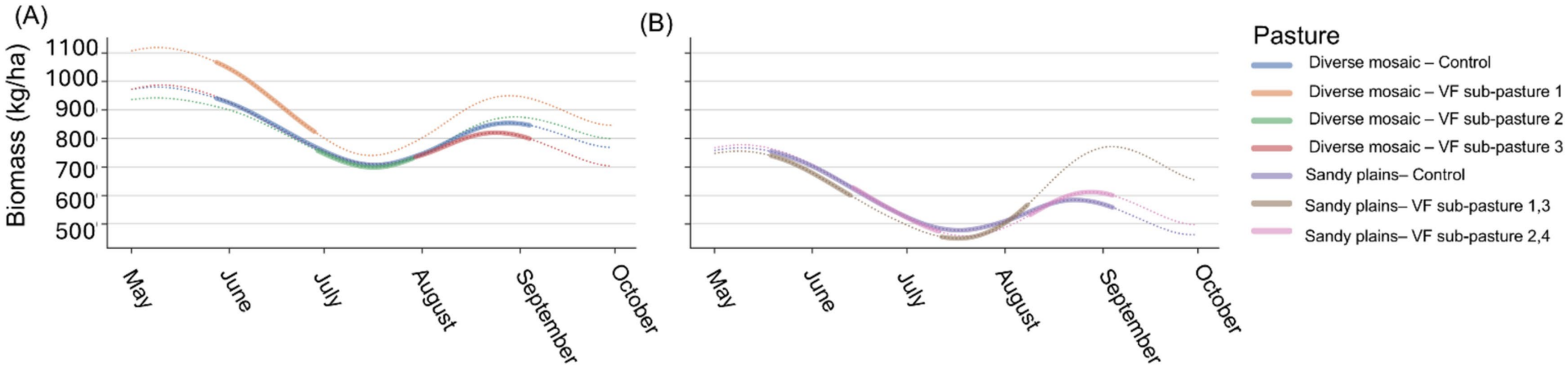
Remotely sensed daily time series of herbaceous biomass (kg/ha) in diverse mosaic pastures, with control and three sub-pastures, showing a general decline and recovery trend **(A)** and Sandy Plains pastures, with control and two sub-pasture groups, also depicting fluctuations **(B)** at the USDA ARS Central Plains Experimental Range, 2024. The legend identifies lines by color. Solid colored lines depict biomass when steers were present in the grazing area, while dotted colored lines show biomass when steers were not present.

We estimated daily diet quality and forage availability for each pasture using satellite-derived maps. Diet quality was represented as dietary crude protein (CP; %) produced following methods described by Kearney, Porensky (55). Forage availability was defined as estimates of total standing herbaceous biomass (kg/ha) produced based on methods described by Kearney, Porensky (56). We refer readers to the original publications for complete details. Briefly, we extracted 30-m resolution gridded surface reflectance data from the Harmonized Landsat-Sentinel (HLS v2.0) for the study area, which we then preprocessed to mask (i.e., remove clouds/shadows), gapfill, and smooth to produce daily time series for a suite of vegetation indices. These indices were then used to predict daily standing herbaceous biomass from an updated version of the model described by Kearney, Porensky (55). Additional phenological metrics were extracted from the Normalized Difference Vegetation Index (NDVI) time series as described by Kearney, Porensky (56) and used in an updated Random Forest model to predict daily dietary CP. We then calculated average daily standing biomass and CP across all grid cells for each pasture area (Figure 1).

### 2.3 Experimental design, instrumentation, and strategy development

This experiment was conducted in four ~130-ha shortgrass steppe pastures from mid-May to early September 2024. A total of 120 British-breed yearling steers, which initially backgrounded in three previous production environments (PPE), were under grazing conditions over a 110-d period. Stocker steer backgrounding is the production stage when individuals are managed from post-weaning until feedlot finishing (57). Two groups of steers originated from Colorado, USA. One group was previously exposed to grazing conditions (Colorado-grazing steers, approximately 13 months of age, *n* = 40, BW 345 kg ± 3.8); in contrast, the other group was initially backgrounded in drylot conditions (Colorado-drylot steers, approximately 12 months of age, *n* = 40, BW 375 kg ± 3.8). The third group originated from Nebraska, USA, where they had previously been exposed to drylot conditions (Nebraska-drylot steers, approximately 11 months of age, *n* = 40, BW 427 kg ± 4.7). A week after arrival in the extensive rangeland grazing environment and collectively grazing a single shortgrass steppe holding pasture (260 ha), approximately 30 steers (*n* = 10 hd per PPE) were randomly allocated to each study pasture, stratified according to their PPE. All cattle in the experiment were fitted with a VF collar (Vence, Merck Animal Health, Rahway, NJ, USA) and weighed prior to entering study pastures. This article utilizes the VF terminology outlined by Ehlert, Brennan (58), maintaining consistency and coherence within the scientific discourse on VF technology.

**Figure 2:**
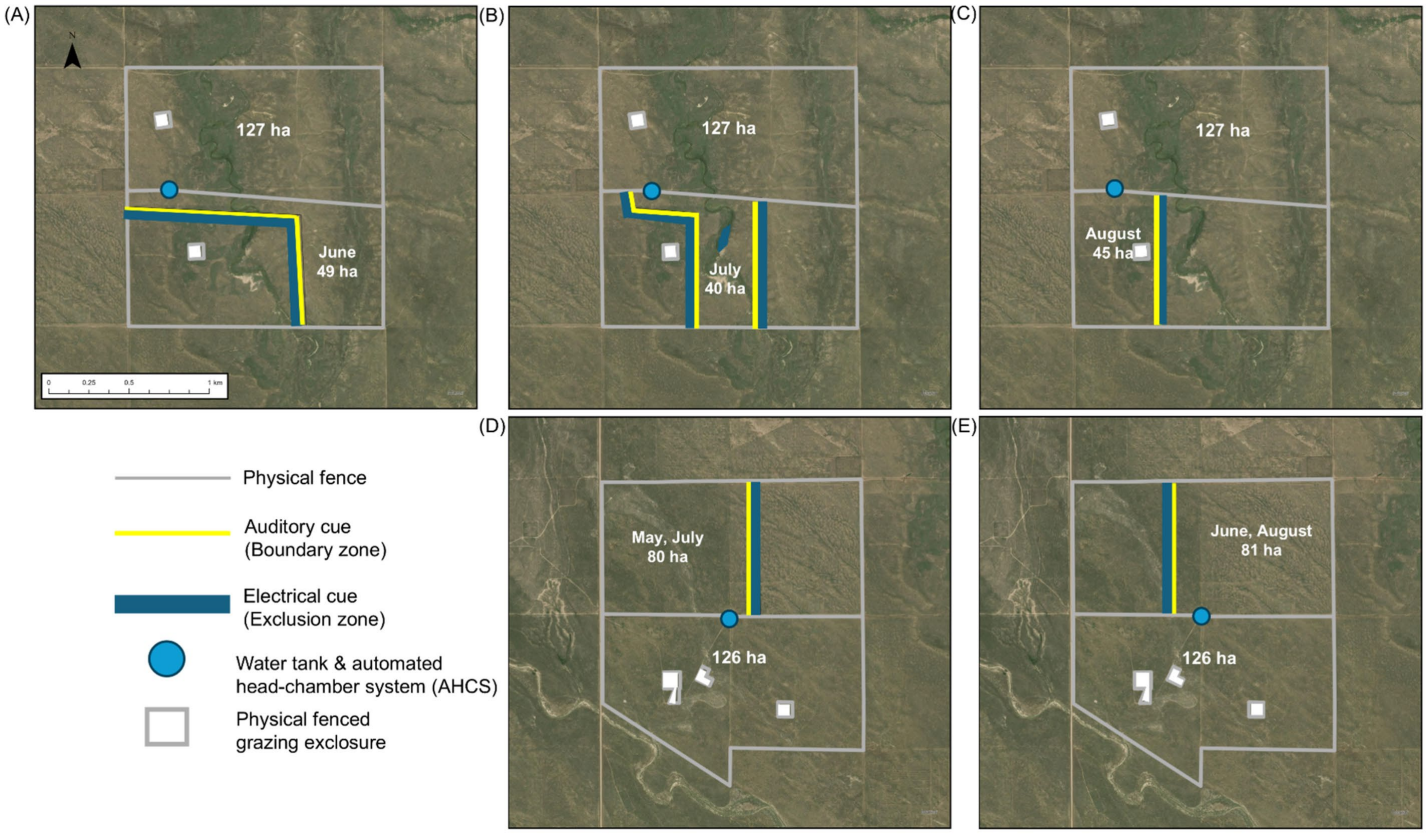
Boundaries of ~130 ha barbed-wire fenced pastures and virtual fence sub-pastures at the USDA ARS Central Plains Experimental Range near Nunn, Colorado, USA. Satellite maps **A–C** depict diverse mosaic pasture-pairs and **D,E** depict Sandy Plains pasture-pair for each rotation interval and hectarage of grazing area, illustrating changes in grazing management.

In one pair, each pasture contained a diverse mosaic of ecological sites consisting of Loamy Plains [ID: R067BY024CO; (59)], Salt Flats [ID: R067BY033CO; (60)], and Sandy Plains [ID: R067BY024CO; (61), Figures 2A–C]. The western third of each pasture in this pair (hereafter, diverse mosaic) consisted of Loamy Plains dominated by the C_4_ shortgrass, blue grama, the central third consisted of Salt Flats dominated by C_4_ midgrasses Alkalai sacaton [*Sporobolus airoides* (Torr.) Torr.] and inland salt grass [*Distichlis spicata* (L.) Greene], and the eastern third contained a mosaic of Loamy and Sandy Plains co-dominated by C_3_ midgrasses western wheatgrass and needle-and-thread, and the C_4_ shortgrass blue grama. In one pasture, VF was used to focus the grazing area on the eastern portion where cool-season grasses were most abundant early in the growing season (May 28 – June 28; Figure 2A). Virtual grazing areas were then shifted to focus the steers onto the Salt Flat portion of the pasture during the middle of the season (June 29 – July 29; Figure 2B), after greenup of the C_4_ warm-season grasses, and then onto the blue grama-dominated Loamy Plains for the last portion of the season (July 30 – September 4; Figure 2C) in an effort to optimize diet quality for the steers across the grazing season. Unlike other assigned grazing areas in the study, the majority of the first and second assigned grazing areas in this VF-managed pasture were 361 and 875 linear m from the pasture’s sole water tank and AHCS (Figures 2A,B).

The second pair of pastures consisted entirely of the Sandy Plains ecological site [ID: R067BY024CO; (61), Figures 2D,E], which was co-dominated by cool-season grasses, primarily western wheatgrass and needle-and-thread, and warm-season grasses, primarily blue grama. In both pastures in this pair, the western half included a shrub layer consisting almost entirely of four-wing saltbush (*Atriplex canescens* [Pursh Nutt.]), which is palatable to cattle, while shrubs were absent from the eastern half. In one of the pastures, VF was used to implement a schedule where the steers grazed the western, shrubland portion of the pasture from May 19 – June 13 (Figure 2D), when the herbaceous layer was starting to green up, and shrubs provided a valuable forage resource for higher diet quality. Steers were then rotated via VF to the eastern half from June 14 – July 11 (Figure 2E), then back to the shrubland from July 12 – August 8 (Figure 2D), and back to the eastern half from August 9 – September 4 (Figure 2E). A key component of this schedule was preventing browsing on the shrubs during the last 3 weeks of August when they are most vulnerable to the impacts of defoliation (62).

These management strategies were developed with a stakeholder group that makes science-informed management decisions during the grazing season (63). Combining high-resolution maps (i.e., sub-meter resolution) of vegetation communities (64) derived from hyperspectral imagery and incorporating local knowledge of the spatial availability and timing of forage resources at the study site played a key role in establishing the grazing area schedule for this 110-d experiment (Table 1).

**Table 1:** Description of the grazing management, remotely-sensed forage quantity and diet quality, and types of plant communities (% of pasture occupied) within the four study pastures and the sub-pastures through which VF-managed steers were rotated, derived from 1 m^2^ resolution hyperspectral imagery.

Ecological site	Treatment	Assigned grazing area	Grazing area (ha)	Date range	Forage availability (kg/ha; mean ± 1 SD)	Dietary crude protein (%; mean ± 1 SD)	Stocking density (steer/ha)	Percentage covered (%)			
								Cool-season forage	Warm-season forage	Cool- and warm-season mix	Bare ground
Diverse mosaic	Virtual fence	17S-1	49	5/28–6/28	952 ± 77	8.5 ± 0.5	0.61	8	53	39	0
		17S-2	40	6/29–7/29	716 ± 17	8.2 ± 0.2	0.75	8	61	30	1
		17S-3	45	7/30–9/4	792 ± 27	7.8 ± 0.3	0.67	7	50	43	0
	Control	17 N-1	127	5/28–6/28	861 ± 53	8.7 ± 0.4	0.24	4	60	35	1
		17 N-2	127	6/29–7/29	724 ± 17	7.9 ± 0.3	0.24	4	60	35	1
		17 N-3	127	7/30–9/4	815 ± 40	7.9 ± 0.2	0.24	4	60	35	1
Sandy Plains	Virtual fence	18S-1	80	5/19–6/13	677 ± 44	8.8 ± 0.5	0.38	43	5	50	2
		18S-2	81	6/14–7/11	545 ± 48	7.9 ± 0.2	0.37	57	9	30	4
		18S-3	80	7/12–8/8	482 ± 37	7.8 ± 0.1	0.38	43	5	50	2
		18S-4	81	8/9–9/4	588 ± 25	7.8 ± 0.3	0.37	57	9	30	4
	Control	19 N-1	126	5/19–6/13	700 ± 39	8.8 ± 0.5	0.24	45	7	45	3
		19 N-2	126	6/14–7/11	546 ± 43	7.9 ± 0.1	0.24	45	7	45	3
		19 N-3	126	7/12–8/8	497 ± 20	7.4 ± 0.1	0.24	45	7	45	3
		19 N-4	126	8/9–9/4	571 ± 12	7.7 ± 0.1	0.24	45	7	45	3

### 2.4 Movement data collection

In this VF system, the end user communicates with a solar-powered base station via a cellular link using the HerdManager software platform. The base station, in turn, uses a VHF radio signal to communicate user-defined coordinates of virtual boundaries and other information to a VF collar worn by the animal. A lithium battery powers the collar and reports animal location at user-defined intervals. Each VF collar has a speaker for auditory cues and metal chain links used as straps that deliver the electrical stimuli. The collar is designed with a weight ballast that keeps the electrical contacts in contact with the animal’s neck; thus, in theory, when the animal receives an electrical stimulus after a series of auditory motivations (65, 66), it turns away from the stimulus, causing the animal to alter its path of travel away from the virtual boundary. In each VF-managed pasture, the training methodology of Boyd, O’Connor (13) was employed for four to 14 d to achieve associative learning between auditory and electrical cues. Steers assigned to control pastures were not enrolled in VF training. VF collars of all animals, both active and inactive, were set to log spatial locations at 5-min intervals. All boundary/exclusion zones for the training period and subsequent grazing area assignments in the management period were created in Vence Herd Manager software (Merck Animal Health, Rahway, NJ, USA).

### 2.5 Animal growth performance and gas flux collection

Body weights (BW) were obtained using a calibrated electronic scale before (d 0) and after the grazing season. Average daily gain (ADG) was calculated for each animal for the study duration (110 d) as the total weight gained (kg/hd) divided by the number of days within the period. A shrink adjustment of 4% was applied to each steer in all BW measurements to estimate shrunk BW (67). Additionally, the VF collar fit was assessed monthly, and adjustments were made as needed (68).

In each pasture, a single pasture-based automated head-chamber system (AHCS) unit (GreenFeed; C-Lock, Inc., Rapid City, SD, USA) was deployed within 3-m of the pasture’s sole water tank to quantify steer enteric gas flux across the grazing season. Before using the AHCS, steers received a radio frequency electronic ID (RFID; Allflex, Madison, WI). Steers were allowed to visit the AHCS every 4 h (up to six visits per day) and consume up to six drops of alfalfa (*Medicago sativa* L.) pellet (approximately 35 g as fed/drop; variation of drop weight was not tracked over season) per visit with 30-s intervals between drops. This schedule encouraged animals to visit the AHCS units throughout the day and ensured they stayed at the AHCS for an appropriate gas collection duration. Recovery tests for CO_2_ were performed monthly, as well as at the beginning and end of the experiment, with recoveries of 100 ± 5%. The manufacturer remotely performed daily zero and span calibrations of the CH_4_ and CO_2_ analyzers via an onboard auto-calibration system. Raw collection data were validated by C-Lock Inc., which included appropriate head proximity, visit length, and airflow and wind corrections, totaling 6,730 records. Data was excluded when the length of the AHCS visit was less than 3 min or greater than 8 min, observations were outside three standard deviations of the mean, and AHCS airflow was less than 26 L/s (69), which resulted in removal of 590 records.

### 2.6 Analytical approach

Location data collected from the VF collars were imported and projected to the WGS 1984 UTM Zone 13 N coordinate system (EPSG: 32613) using package ‘sf’ (70). Because the VF collars sometimes logged GPS fixes at frequencies below 5 min when animals are near a virtual fence, we screened the dataset to remove high-frequency fixes (i.e., retaining only one fix per five-minute interval) prior to analysis. To evaluate the efficacy of the VF system in terms of containing steers within the desired grazing areas, we sought to calculate the frequency at which individuals moved through the boundary/exclusion zone. One challenge in calculating breaches from the grazing area is that individuals located near the boundary/exclusion zone but still within the desired grazing area often have multiple GPS locations that occur outside the grazing area, simply due to GPS error. We developed a process to screen a large GPS location dataset, differentiating between breaches of grazing areas by a steer and GPS errors. First, we identified all locations outside the grazing area for each individual and date for a given VF assignment. We then calculated the distance from each ‘outside’ location to the nearest assigned boundary using the ‘*near*’ tool in ArcGIS Pro v3.5, and calculated the mean distance, maximum distance, and number of locations outside the grazing area for each steer per day. When we checked movement pathways visually in ArcGIS Pro v3.5, we found that steer-days with 10 or fewer locations outside the grazing area or a mean distance from the nearest grazing area boundary less than 22-m were consistently associated with a pattern that appeared to be due to GPS error (e.g., locations alternating between out and in the VF paddock but staying in the same general vicinity). In contrast, days with more than 10 locations outside and mean distance to the nearest grazing area greater than 22-m were consistently associated with a pattern of moving through the boundary/exclusion zone, traveling in a loop outside the grazing area for multiple consecutive locations, and then returning toward the grazing area. We used this screening process to identify all steer-days where VF-managed steers moved out of the VF grazing area and calculated the percent of all GPS fixes collected for a given herd and rotation interval that resulted from excursions outside the assigned grazing area.

To make inferences about spatial behavior due to VF management in relation to growth performance and enteric CH_4_ emissions, we first conducted analyses using only steers that met the AHCS-visitation requirements (Table 2), and then repeated analyses for all steers regardless of AHCS visitation status. Using the projected GPS location data, the daily distance traveled per steer (m/hd/d) was calculated using the ‘*st_distance*’ function, which applied the Pythagorean theorem to sequential GPS coordinates within a day. To assess exploration area in response to VF management, the daily area explored (ha/hd/d) was calculated using the *‘st_area’* function for each individual, which generated a polygon encompassing the minimum area containing all GPS coordinates recorded within a given day.

**Table 2:** Automated head-chamber system (AHCS) visitation statistics (mean ± SE) for growing steers under extensive grazing conditions at the USDA Central Plains Experimental Range in northeatern Colorado, 2024.

Ecological Site	Data set	Item	VF-status	
			VF-managed	Control
Diverse mosaic	AHCS-acclimated	# of AHCS eligible steers	9	6
		# of AHCS visits per day per eligible steer	0.75 ± 0.01	0.70 ± 0.01
		# of AHCS visits per rotation per eligible steer	24.9 ± 0.5	22.3 ± 0.3
		# of AHCS eligible steers per rotation	7.3 ± 1.4	4.7 ± 0.3
	All^1^	# of steers	28	30
Sandy Plains	AHCS-acclimated	# of AHCS eligible steers	17	22
		# of AHCS visits per day per eligible steer	1.02 ± 0.01	0.81 ± 0.01
		# of AHCS visits per rotation per eligible steer	22.1 ± 0.2	27.4 ± 0.2
		# of AHCS eligible steers per rotation	12.0 ± 1.1	18.8 ± 0.4
	All	# of steers	30	30

AHCS eligible steers are individuals who visited the AHCS unit at least 15 times in a minimum of each of two rotation intervals of the three or four rotation intervals depending on diverse mosaic and Sandy Plains pasture-pair, respectively, across the grazing season.

1 Two individuals could not be included in the further analysis due to the lack of an endweight for calculating growth performance.

Initial growth performance and enteric emissions data processing were done in RStudio v 12.1.563 (71) with the ‘tidyverse’ package (72). ArcGIS Pro v3.5 (73) was used to visualize and screen GPS collar data. For each period in which a given virtual fence boundary was in use (i.e., for each rotation interval within a given pasture pair), we examined the effect of VF management on daily distance traveled, area explored, and enteric CH_4_ emissions. Using the ‘nlme’ package (74), linear mixed models with rotation interval as a repeated measure and VF status (i.e., VF-managed or control) as a fixed effect and the animal’s PPE (50) and a unique animal identifier as random intercepts were employed to assess animal spatial behavior, growth performance, and enteric CH_4_ emissions variation as the season progressed. A first-order autoregressive autocorrelation structure was included for repeated measures on each steer. The model structure that best fit the data was selected according to Schwartz’s Bayesian information criterion. Note that these within-season analyses did not include evaluation of ADG because we only weighed steers at the beginning and end of the full grazing season.

We also conducted analyses of ADG (kg/hd/day), enteric CH_4_ emissions (g/hd/day), and CH_4_ emissions intensity (g CH_4_ emitted/kg of weight gained) averaged over the entire grazing season as response variables, and VF status, block of the experiment (diverse mosaic vs. Sandy Plains) and the steer’s PPE and their interactions as fixed effects. We also examined the relationship between ADG and enteric CH_4_ emissions at the individual steer level via a simple linear regression. We present this regression graphically with individual steers colored by PPE and by VF treatment/block combination to illustrate how these factors all covary. In these analyses, we only used steers that met AHCS-visitation requirements of at least 15 AHCS visits of a minimum of 3-min duration per individual steer (75) in a minimum of two of the three or four rotation intervals in the study pastures. A separate analysis of variance was performed for ADG of all study steers, irrespective of AHCS-visitation requirement status. Means were assumed to be significantly different at *p* ≤ 0.05 and tendencies at 0.05 < *p* ≤ 0.10. We acknowledge that this study was not replicated at the pasture level, hence our statistical inferences regarding spatial behavior, CH_4_ emissions, and ADG are restricted to the specific conditions of the study pastures.

## 3 Results

### 3.1 Spatial distribution management evaluation

#### 3.1.1 Diverse mosaic pasture pair

**Figure 3:**
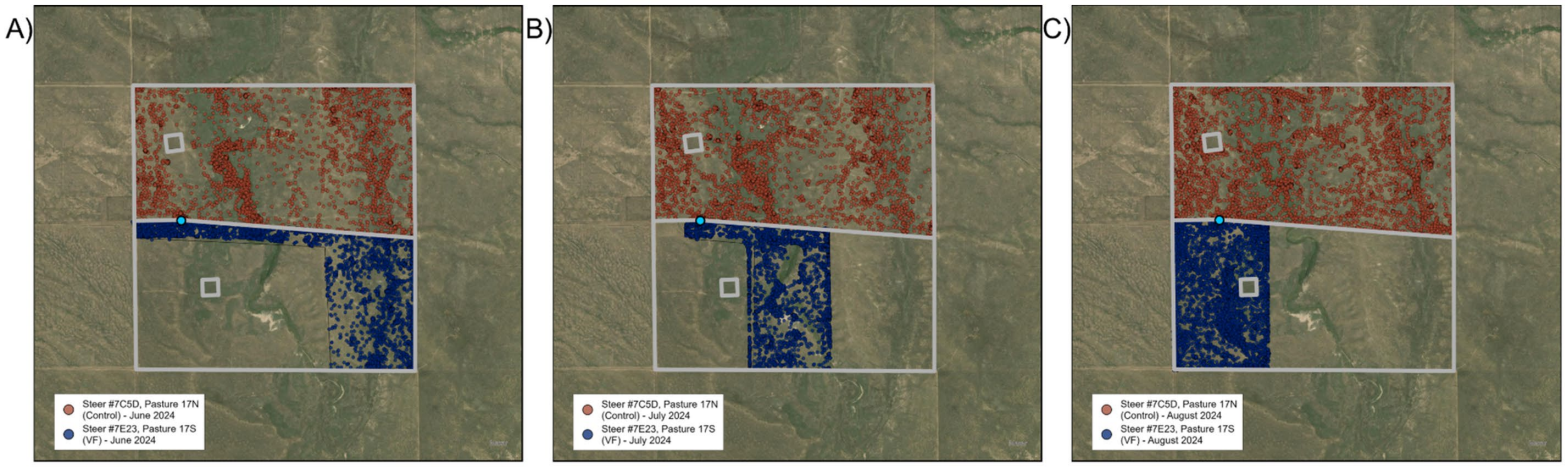
Satellite maps of locations of virtual fence-managed steer #7E23 (blue dots) and control steer #7C5D (red dots) across the three rotation intervals **(A–C)** in VF-managed and control pastures of the diverse mosaic pasture pair. Light blue circle denotes location of water tank and automated head-chamber system (AHCS) location.

During the first rotation interval, the June assignment, two individuals pushed through the barbed wire fence (north side) on the first day of the assignment, were herded back into the desired grazing area on day three and then remained in the grazing area for the remainder of the June assignment. Four steers moved through the VF boundary (west and south side) on day four, spent most of the day outside the desired grazing area, and then returned to the desired grazing area. Each day thereafter, these four steers left the assigned grazing area, spent several hours outside the VF often including a visit to a permanent pond (Figure 3A), and then returned into the desired grazing area to rejoin the herd. A fifth steer breached on the 18^th^ day of the June assignment and then continued to do so for the remainder of the period. Because these five steers still spent most of their time within the desired grazing area, the vast majority of the herd’s time was still spent within the desired grazing area. For the June assignment, we found that 9.7% of all GPS locations were located outside the desired grazing area. After excluding locations identified as GPS errors, 5.1% of all locations occurred outside the desired grazing area as a result of breaches of the boundary/exclusion zone.

During the second rotation interval, the July assignment (Figure 3B), the same five steers continued to make daily breaches; one of these five began noncompliance behavior on day one, three started on day two, one on day four, and the fifth individual on day five. In addition, four other steers made excursions outside the assigned grazing area on a single day, but after returning to the grazing area never repeated the noncompliance behavior. For the July assignment, we found that 9.7% of all GPS locations were positioned outside the desired grazing area, and that 6.1% of all locations were outside due to breaches.

For the third rotation interval, the August assignment (Figure 3C), the same five steers again made daily excursions outside their desired grazing area, in this case with two starting on day two, two on day 22, and one on day 27. A sixth steer breached and then returned into the grazing area on day 30 and continued to do so daily thereafter. Two steers breached and then returned into the grazing area on a single day, and then never repeated the noncompliance behavior. Three individuals exhibited noncompliance behavior on 2 or 3 days, then did not repeat. In the August assignment, we found that 6.4% of all GPS locations were located outside the grazing area, and that 3.6% were outside due to breaches.

In the continuous grazing diverse mosaic pasture (i.e., the control pasture), where non-active VF collars were deployed to collect GPS location data, one individual on day 10 breached and was returned within that day, and the herd breached for 2 days from day 14 to 16. Over the entire grazing season, we found that 0.07% of all GPS locations were outside the fence, and after correcting for GPS error, <0.01% were outside due to breaches.

#### 3.1.2 Sandy Plains pasture pair

**Figure 4:**
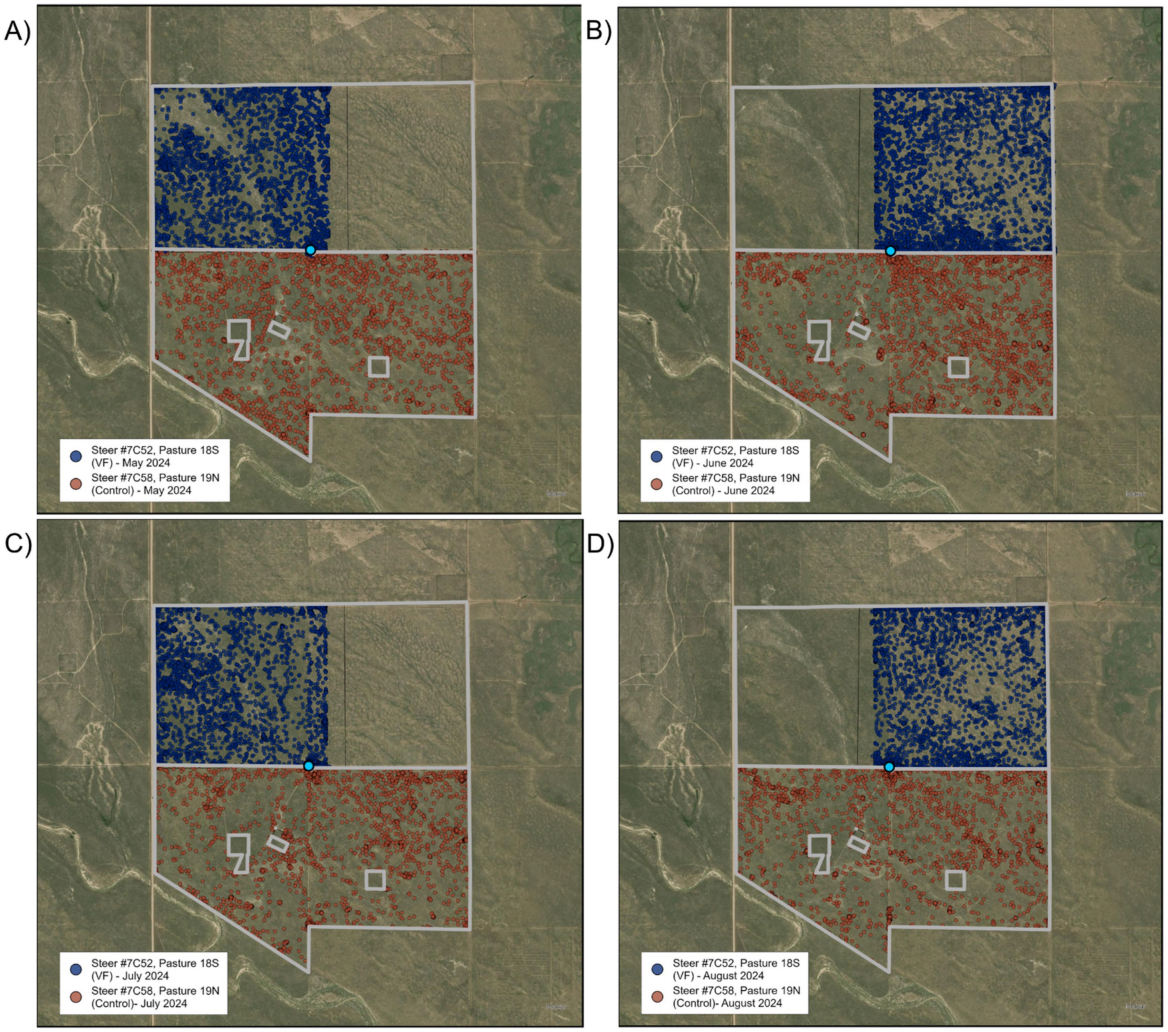
Satellite maps of locations of virtual fence-managed steer #7C52 (blue dots) and control steer #7C58 (red dots) across the four rotation intervals **(A–D)** in VF-managed and control pastures of the Sandy Plains pasture pair. Light blue circle denotes location of water tank and automated head-chamber system (AHCS) location.

During the first rotation interval, the cattle were restricted to the western half of the pasture via a boundary/exclusion zone running north to south near the center of the allotment (Figure 4A). On the second day of this grazing area assignment, one steer moved through the boundary/exclusion zone, remained outside of it for approximately 1 h, then rejoined the herd inside the grazing area. On the seventh day, the majority of the herd (21 of the 30 steers) moved through the boundary/exclusion zone and spent an average of 3.1 h outside of it before returning back to the grazing area. The steers appeared to have been resting (i.e., stationary) near the corner of the grazing area when the herd suddenly moved through it, all together at the same time, suggesting they were startled by an unknown entity. This behavior never occurred again during this assignment. On the 13th day of the first rotation interval, one steer moved through the boundary/exclusion zone, spent ~2.5 h outside, and then rejoined the herd. On the 22nd day, another steer moved through the boundary/exclusion zone and remained outside for ~30 min before rejoining the herd. We found that 4.4% of all GPS locations were located outside the desired grazing area, but after removing those associated with GPS error, only 0.69% were outside due to boundary/exclusion zone breaches.

During the second rotation interval (Figure 4B), movements through the boundary/exclusion zone were rare, with a single steer moving out during the first day, and another moving out during the last day. After correcting for GPS error, we found that steers spent less than 0.05% of their time outside the desired grazing area. Results were similar for the July assignment (Figure 4C), with steers spending less than 0.05% of their time outside the desired grazing area. During the August assignment (Figure 4D), excursions outside the boundary/exclusion zone became more frequent, with three steers beginning to breach the boundary/exclusion zone on days three, seven, and eight, and continuing to do so daily thereafter. After correcting for GPS error, 2.2% of the GPS locations were outside the desired grazing area.

In the continuous grazing Sandy Plains pasture, where non-active VF collars were deployed to collect GPS location data, three individuals left the pasture for collar adjustments for 2 days, and one individual breached the fixed barbed-wire fence on four different occasions but was returned within the same day. Over the course of the entire grazing season, we found that 0.11% of all GPS locations fell outside the pasture fence, and that after corrected for GPS error, < 0.01% of locations occurred outside the pasture fence.

### 3.2 Spatial behavior evaluation

#### 3.2.1 Diverse mosaic pasture pair

**Figure 5:**
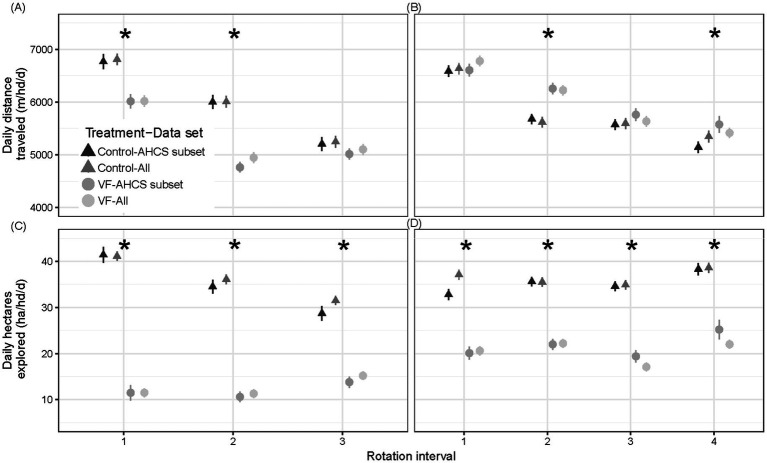
Comparisons of mean ± SE daily distance traveled (m/hd/d) and daily area explored (ha/hd/d) for VF-managed and control steers over the study period in diverse mosaic-associated pastures **(A,C)** and Sandy Plains ecological site-associated pastures **(B,D)**. Mean ± SE for automated head-chamber system (AHCS)-acclimated and all steers are depicted. Asterisks denote significance at *p* < 0.05 for means comparison of AHCS-acclimated steers; see text for all steer results.

A significant VF status × rotation interaction (*p* < 0.0001) for the daily distance traveled demonstrated that VF-managed AHCS-acclimated steers traveled less than control AHCS-acclimated steers in June (6,015 m/hd/d ± 134 vs. 6,768 m/hd/d ± 141, *p* = 0.002) and July (4,762 m/hd/d ± 100 vs. 6,001 m/hd/d ± 130, *p* < 0.0001) but no difference was found in August (5,205 m/hd/d ± 131 vs. 5,015 m/hd/d ± 105, *p* = 0.28; Figure 5A).

The same outcome was determined for all study steers, irrespective of AHCS-acclimation status. A significant VF status × rotation interaction (*p* < 0.0001) for the daily distance traveled demonstrated that all VF-managed steers traveled less than all control steers in June (6,019 m/hd/d ± 105 vs. 6,811 m/hd/d ± 110, *p* = 0.002) and July (4,946 m/hd/d ± 105 vs. 6,007 m/hd/d ± 113, *p* < 0.0001) but no difference was found in August (5,248 m/hd/d ± 110 vs. 5,103 m/hd/d ± 103, *p* = 0.34).

In addition, although the daily area explored by AHCS-acclimated VF-managed steers was less than that of AHCS-acclimated control steers for each rotation interval (grand mean ± SE: 12.0 ha/hd/d ± 0.80 vs. 34.9 ha/hd/d ± 0.92, VF status *p* < 0.0001; Figure 5C), a VF status × rotation interval interaction (*p* < 0.0001) showed that the daily area explored by AHCS-acclimated control steers decreased as the season progressed (41 ha/hd/d ± 1.7 to 29 ha/hd/d ± 1.6, month *p* ≤ 0.02), while the area explored by AHCS-acclimated VF-managed steers did not decrease (11.5 ha/hd/d ± 1.7 to 13.8 ha/hd/d ± 1.2; month *p* ≥ 0.13). A similar outcome was determined for all study steers, irrespective of AHCS-acclimation status. Daily area explored by all VF-managed steers was less than that of AHCS-acclimated control steers for each rotation interval (grand mean ± SE: 12.7 ha/hd/d ± 0.54 vs. 36.2 ha/hd/d ± 0.59, VF status *p* < 0.0001; Figure 5C), a VF status × rotation interval interaction (*p* < 0.0001) showed that the daily area explored by AHCS-acclimated control steers decreased as the season progressed (41 ha/hd/d ± 1.01 to 32 ha/hd/d ± 0.96, month *p* ≤ 0.004), while the area explored by AHCS-acclimated VF-managed steers increased as the season progressed (11.5 ha/hd/d ± 0.96 to 15.2 ha/hd/d ± 0.90; month *p* ≤ 0.01) apart from the June to July intervals (11.5 ha/hd/d ± 0.96 vs. 11.3 ha/hd/d ± 0.96, *p* = 0.98).

#### 3.2.2 Sandy Plains pasture pair

A VF status x rotation interval interaction (*p* = 0.0004) for AHCS-acclimated steers indicated that the effect of VF management on daily distance traveled depended on rotation interval (Figure 5B). During May and July, AHCS-acclimated steers managed with and without VF traveled similar distances per day (May: 6605 m/hd/d ± 121 vs. 6,588 m/hd/d ± 105, *p* = 0.91, July: 5761 m/hd/d ± 117 vs. 5,574 m/d ± 98, *p* = 0.23). In contrast, AHCS-acclimated VF-managed steers traveled a greater distance per day than control steers in June and August rotation intervals (June: 6256 m/d ± 110 vs. 5,679 m/d ± 96, *p* = 0.0003, August: 5576 m/hd/d ± 157 vs. 5,144 m/hd/d ± 109, *p* = 0.03). For AHCS-acclimated steers, the daily distance traveled by VF-managed steers was greater than control steers (VF status grand mean: 6049 m/hd/d ± 97 vs. 5,746 m/hd/d ± 83, *p* < 0.0001; Figure 5B); and, travel distance generally decreased from rotation interval to interval both with and without VF-management (Figure 5). Daily travel distance of AHCS-acclimated VF-managed steers decreased over the season (6,605 m/hd/d ± 121 to 5,576 m/hd/d ± 157, *p* ≤ 0.01), apart from July to August rotation intervals (5,761 m/hd/d ± 117 vs. 5,576 m/hd/d ± 157, *p* = 0.60), while travel distance also decreased over the season control steers (6,588 m/hd/d ± 105 to 5,144 m/hd/d ± 109, *p* < 0.0001), apart from June to July rotation intervals (5,679 m/d ± 96 vs. 5,574 m/d ± 98, *p* = 0.62). Additionally, for AHCS-acclimated steers the daily area explored was less for VF-managed than control steers (22 ha/hd/d ± 0.76 vs. 35 ha/hd/d ± 0.58; VF Status *p* < 0.0001), which did not depend on the rotation interval (VF status × rotation interval: *p* = 0.76; Figure 5D).

A similar outcome was determined for all study steers in the Sandy Plains pasture-pair, irrespective of AHCS-acclimation status. A VF status x rotation interval interaction (*p* < 0.0001) for all steers indicated that the effect of VF management on daily distance traveled depended on rotation interval. While in the grazing area assignments for May, July, and August, all steers managed both with and without VF traveled similar distances per day (May: 6779 m/hd/d ± 99 vs. 6,637 m/hd/d ± 107, *p* = 0.33, July: 5635 m/hd/d ± 97 vs. 5,595 m/hd/d ± 105, *p* = 0.77, August: 5357 m/hd/d ± 109 vs. 5,414 m/hd/d ± 98, *p* = 0.69). In contrast, all VF-managed steers traveled a greater distance per day than all control steers in the June rotation interval (June: 6225 m/hd/d ± 96 vs. 5,619 m/hd/d ± 103, *p* = 0.0001). The daily distance traveled by all VF-managed steers was marginally greater than by all control steers (VF status grand mean: 6014 m/hd/d ± 97 vs. 5,802 m/hd/d ± 86, *p* = 0.08). Travel distance generally decreased from rotation interval to interval for both control and VF-managed steers (Figure 5). In addition, the daily area explored was less for all VF-managed than all control steers (21 ha/hd/d ± 0.49 vs. 36.4 ha/hd/d ± 0.54; VF status *p* < 0.0001), which did not depend on the rotation interval (VF status × rotation interval: *p* = 0.12).

### 3.3 Enteric CH_4_ emissions evaluation by rotation interval

#### 3.3.1 Diverse mosaic pasture pair

**Figure 6:**
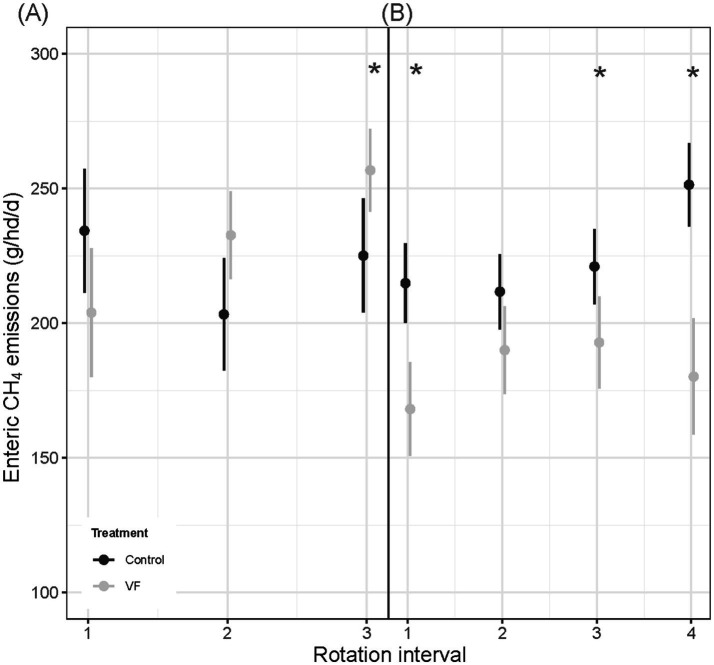
Comparisons of mean ± SE enteric CH_4_ emissions (g/hd/d) for VF-managed and control steers over the study period in diverse mosaic-associated pastures **(A)** and Sandy Plains ecological site-associated pastures **(B)**. Asterisks denote significance at *p* < 0.05 for means comparison.

Of the 15 individuals who met AHCS-visitation requirements across the diverse mosaic pasture pair, nine steers were managed with VF, while six steers were not VF-managed (Table 2). A significant VF status × rotation interval interaction (*p* = 0.001; Figure 6A) for enteric CH_4_ production revealed that the effect of VF status on CH_4_ production was not consistent over the progression of rotation intervals. VF-managed steers tended to produce less CH_4_ than control steers in the June interval (204 g/hd/d ± 12.23 vs. 234 g/hd/d ± 11.70, *p* = 0.10), while control steers emitted less CH_4_ than VF-managed steers in the July interval (203 g/hd/d ± 10.6 vs. 233 g/hd/d ± 8.3, *p* = 0.05) and the August interval (225 g/hd/d ± 10.8 vs. 257 g/hd/d ± 7.8, *p* = 0.03). A marginal main effect of VF status (*p* = 0.08) indicated that control steers emitted less CH_4_ than VF-managed steers (221 g/hd/d ± 8.58 vs. 231 g/hd/d ± 7.24), while a significant rotation interval effect (*p* = 0.0001) showed that CH_4_ emissions increased as the season progressed for VF-managed steers (204 g/hd/d ± 12.2 to 257 g/hd/d ± 7.8, *p* ≤ 0.05). Enteric CH_4_ emissions of control steers did not increase over the season (*p* ≥ 0.13).

#### 3.3.2 Sandy Plains pasture pair

In the Sandy Plains-dominated pasture pair, a total of 39 AHCS-acclimated steers met the visitation requirements and were used in subsequent gas flux analysis. Out of the 39 AHCS-acclimated steers, 17 individuals were VF-managed, while 22 individuals were not managed with VF (Table 2). A significant VF status × rotation interval interaction (*p* < 0.0001; Figure 6B) for enteric CH_4_ production revealed that VF-managed steers produced less CH_4_ than control steers in May (168 g/hd/d ± 8.9 vs. 215 g/hd/d ± 7.5, *p* = 0.0003), July (193 g/hd/d ± 8.7 vs. 221 g/hd/d ± 7.1, *p* = 0.06), and August (180 g/hd/d ± 11.0 vs. 251 g/hd/d ± 8.0, *p* < 0.0001), while a tendency was observed for June (190 g/hd/d ± 8.32 vs. 212 g/hd/d ± 7.12, *p* = 0.06). The main effect of VF status (*p* < 0.0001) indicated that VF-managed steers emitted less CH_4_ than control steers (183 g/hd/d ± 7.78 vs. 225 g/hd/d ± 6.65, *p* < 0.0001), while a rotation interval main effect (*p* < 0.0001) showed that CH_4_ emissions increased as the season progressed for control steers (215 g/hd/d ± 7.5 to 251 g/hd/d ± 7.9, *p* < 0.0001). In contrast, a seasonal increase in CH_4_ emissions for VF-managed steers was not observed (*p* = 0.63).

### 3.4 Season-long growth performance, enteric CH_4_ emissions, and enteric CH_4_ intensity

**Figure 7:**
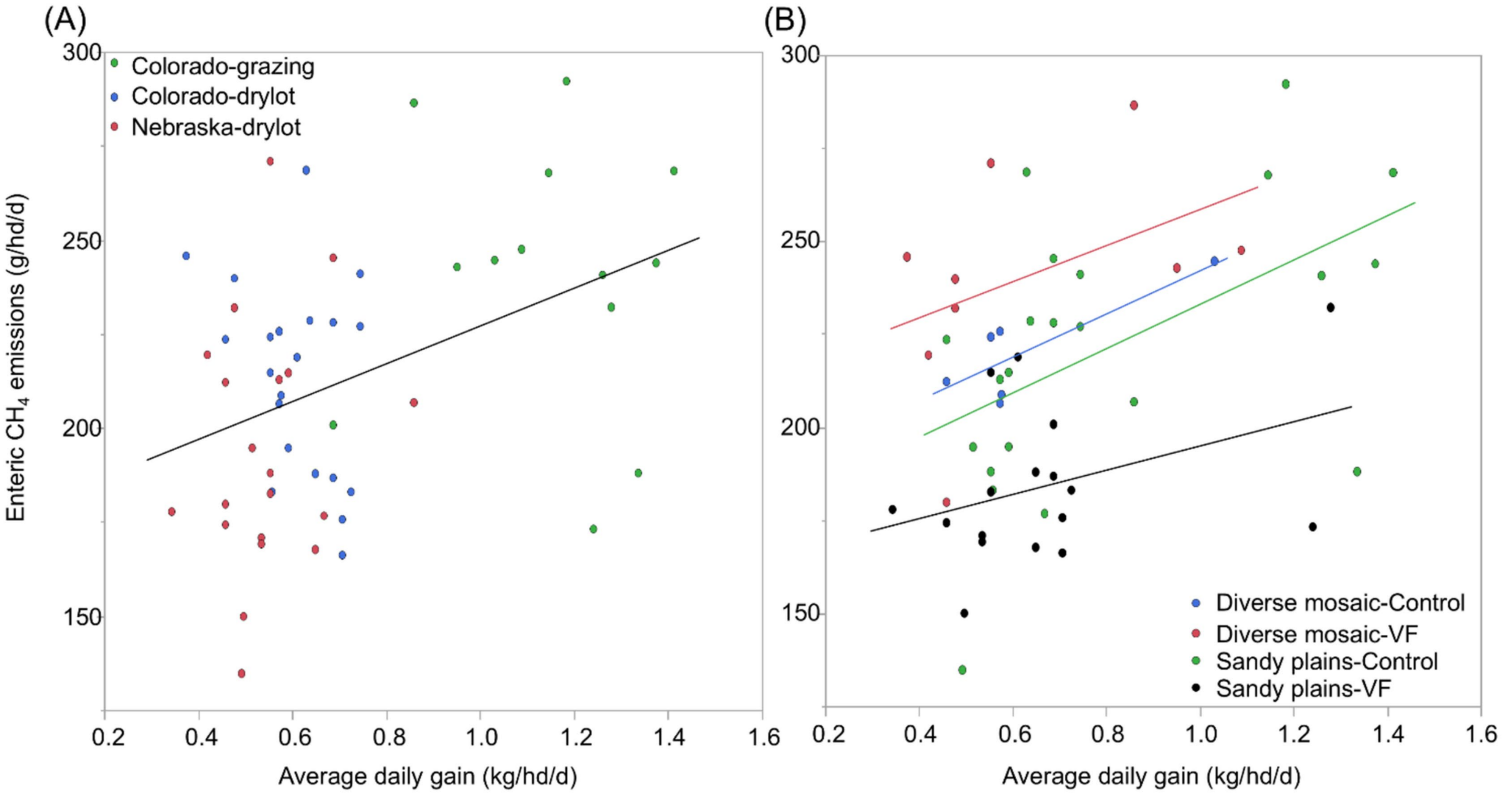
Variation in average daily gain (kg/hd/d) and enteric CH_4_ emissions (g/hd/d) of yearling steers in relation to their previous production environment **(A)** and management with vs. without virtual fence **(B)** in the shortgrass steppe of northeastern Colorado.

Across all four pastures, we obtained season-long estimates of both ADG and CH_4_ emissions for 54 steers (48% of 120 hd). For these 54 individuals, ADG averaged 0.71 kg/hd/d (1 SD = 0.28) and CH_4_ emissions averaged 213 g/hd/d (1 SD = 35.5; Table 3), such that ADG was twice as variable among individuals (CV = 39.3%) as CH_4_ emissions (CV = 16.7%). After accounting for variation in sample size among individuals from the 3 different PPE’s, the overall least-square mean for CH_4_ emissions was 218 g/hd/d (Table 3). We found that ADG was positively but weakly related to CH_4_ emissions (CH_4_ = 176.8 + 50.2 x ADG; R^2^ = 0.16, F_1,52_ = 9.72, *p* = 0.003; Figure 7A). When we examined a model of individual ADG as a function of PPE, VF status, and pasture type and their interactions, we found no significant interactions (*p* > 0.15). ADG varied most strongly among PPE (*p* < 0.0001), with model-predicted, least-square means (± 1SE) of 0.51 + 0.03, 0.58 ± 0.03, and 1.11 + 0.03 kg/hd/day for Nebraska-drylot, Colorado-drylot, and Colorado-grazing steers, respectively (Figure 7A). ADG was 0.12 kg/hd/day lower in the diverse mosaic compared to the Sandy Plains block (*p* = 0.01) and was reduced by 0.07 kg/hd/day for VF-managed compared to control steers (*p* = 0.03; Table 3). Enteric CH_4_ emissions exhibited a pasture type x VF status interaction (*p* = 0.003) because (1) emissions were similar in both control pastures, (2) showed a marginal increase with VF management in the diverse mosaic block (+19 g/hd/day; *p* = 0.096), and (3) decreased by 34 g/hd/day with VF management in the Sandy Plains block (*p* = 0.001; Table 3). Enteric CH_4_ emissions also varied significantly with PPE (*p* = 0.001), averaging 198 ± 7, 218 ± 6 and 239 ± 8 for Nebraska-drylot, Colorado-drylot, and Colorado-grazing steers, respectively (Figure 7A). Enteric CH_4_ emissions intensity varied most with PPE (*p* < 0.001), averaging 383 ± 19, 395 ± 17 and 217 ± 21 g CH_4_/kg of daily weight gain for Nebraska-drylot, Colorado-drylot, and Colorado-grazing steers, respectively. For emissions intensity, we also found a significant block × VF status interaction (*p* = 0.001), as it was unaffected by VF status in the Sandy Plains block (*p* = 0.47) and increased by 106 g CH_4_/kg in the diverse mosaic block (*p* = 0.011; Table 3). The lower CH_4_ emission intensity for Colorado-grazing steers reflects their greater ADG and only slightly greater CH_4_ emissions compared to the steers backgrounded in drylot environments (Figure 7A). Analysis of ADG for all steers in the experiment revealed similar results as the analysis of the subset of AHCS-acclimated steers. ADG for all steers was most strongly affected by PPE (*p* < 0.001), with additional significant effects of both VF status (*p* = 0.004) and pasture type (*p* < 0.001; Table 3; Figure 7B).

**Table 3:** Least-square mean estimates and standard errors for average daily weight gain, enteric CH_4_ emissions, and the ratio of enteric CH_4_ emissions to weight gain for yearling steers managed with and without virtual fence on two different pasture types and overall pastures at the Central Plains Experimental Range in northeastern Colorado, averaged over the full grazing season in 2024.

Data set	Variable	Diverse mosaic		Sandy Plains		Both pastures combined		Overall
		VF	Control	VF	Control	VF	Control	
AHCS-acclimated (*n* = 54)	Average daily gain (kg/hd/d)	0.64 ± 0.04	0.73 ± 0.05	0.75 ± 0.03	0.84 ± 0.02	0.69 ± 0.03	0.78 ± 0.03	0.72 ± 0.03
	CH_4_ production (g/hd/d)	241 ± 9	223 ± 12	187 ± 7	223 ± 5	216 ± 5	221 ± 6	218 ± 8
	CH_4_ intensity (g CH_4_/kg ADG)	431 ± 23	324 ± 31	278 ± 18	294 ± 14	352 ± 14	318 ± 16	335 ± 14
All (*n* = 118)	Average daily gain (kg/hd/d)	0.60 ± 0.02	0.64 ± 0.02	0.77 ± 0.02	0.87 ± 0.02	0.69 ± 0.02	0.75 ± 0.02	0.72 ± 0.03

Automated head-chamber system (AHCS)-acclimated steers were those with sufficient number of visits to the AHCS to estimate methane emissions.

## 4 Discussion

We evaluated the efficacy of implementing rotational grazing management via VF technology in terms of its influence on steer spatial behavior and the consequences for both animal growth and CH_4_ emissions. Overall, the VF system was successful in manipulating steer distribution as planned in the within-pasture rotational treatments. In one block where five of 30 steers often moved through the virtual fence boundaries on a daily basis, the herd still collectively spent 94–96% of their time within the desired grazing areas. In the second block, excursions through the VF boundary were rare, and collectively the herd spent more than 98% of their time within the desired grazing areas. The successful implementation of the grazing rotation via VF was reflected in patterns of forage biomass accumulation, with rested portions of the VF-managed pastures increasing in forage biomass much more rapidly than the full paddock being grazed following rains in the second half of the growing season (Figures 1A,B). One key result was that VF-management consistently reduced steer weight gain across both pasture types (by 12% for ACHS-acclimated steers and 9% for all steers in the experiment). For the grazing season, this translated into a 10.2 kg (22.4 lb) lower weight than control steers at the end of the grazing season. These findings are consistent with prior rotational grazing experiments at the study site which employed larger herds rather than subdividing pastures (4, 19). The increase in stock density of the VF-managed steers may have induced less selective grazing, as the sub-pastures were more homogenous in plant community composition than the controls.

In terms of the consequences for enteric CH_4_ emissions, VF-managed steers in the diverse mosaic pasture-pair produced more CH_4_ when grazing C_4_ grass-dominated communities during the latter two-thirds of the grazing season and produced less CH_4_ than controls while grazing a cool-season dominated sub-pasture early in the season. Averaged over the entire season, no differences were observed in CH_4_ emissions, but VF-managed steers emitted 33% more CH_4_ per kg of weight gain than controls. In the Sandy Plains pair of pastures, VF-managed steers produced less CH_4_ than control steers in all four rotations, all involving grazing of cool-season dominated sub-pastures. However, this outcome occurred at the cost of a 12% decrease in kg gained per day, resulting in no differences in CH_4_ emission intensity between VF versus control steers over the whole season. The variability in the direction and magnitude of differences in CH_4_ emission rates across pastures and plant communities suggests that concentrating grazing on cool-season forage may be more likely to reduce enteric emissions, while the opposite is true for targeted grazing of warm-season forage. At the same time, a reduction in weight gain associated with targeted grazing can result in an overall increase in CH_4_ emission intensity, which is ultimately the key metric for sustainable intensification of food production.

Our findings are viewed from the subset of animals that routinely used the AHCS across all pastures; therefore, conclusions about study treatments and enteric emissions from this unique empirical study must be drawn with caution. Nonetheless, our coupling of animal GPS tracking and AHCS data indicated that differences in spatial behavior were consistent between VF-managed and control steers irrespective of their AHCS-acclimation status. This finding supports the view that the subset of steers that were acclimated to the AHCS in each treatment were representative of the wider herd population. Such fusion of animal performance and enteric emissions information with movement behavior enhances our confidence in estimates of enteric CH_4_ emission in extensive rangeland settings.

Additionally, we note that all analyses were conducted at the individual steer level, such that inferences are restricted to these specific pastures and weather conditions, and additional replication of VF treatments at the pasture level and seasons of study are needed to test the broader implications for ADG and CH_4_ emissions in semiarid rangelands. However, issues related to spatial behavior under distinct types of rangeland management have not been considered before in enteric CH_4_ studies. Thus, our experiment illustrates that multiple factors have potential impact on the management of the forage resource-growth performance-enteric emissions relationship and can contribute to reducing uncertainty around the development of sustainable grazing practices for extensive livestock farming.

Steers in this study were obtained from three different previous production environments (PPE). Interestingly, steers from these different sources differed substantially in their growth performance on shortgrass rangeland, with steers that spent the previous winter grazing shortgrass achieving significantly greater weight gains than steers backgrounded in drylots. Further, steers of different origins differed to only a limited degree in CH_4_ emissions, such that the steers backgrounded on rangeland had significantly lower CH_4_ emissions per kg of beef produced compared to steers from drylot origin. These three different groups of steers also differ in terms of their genetics; thus, we cannot determine whether background, genetics, or other factors are driving the differences in ADG. However, we can conclude that factors other than grazing management during the growing season can have large effects on ADG and CH_4_ emission intensity, thereby muting the effects of grazing management.

Due to the greater quantity and quality of forage resources, shortgrass steppe pastures associated with the Sandy Plains ecological site (76), were expected to support enhanced steer growth performance (77) and concomitantly reduced enteric CH_4_ emissions when herd distribution focused on available cool-season grass swards. This expectation was largely met, as pooled weight gains were 24% greater on the Sandy Plains pastures compared to the diverse mosaic pastures. However, differences in CH_4_ emissions between the pasture types depended on spatial management, with both pasture types having the same CH_4_ emission rates in the absence of VF management but 25% less emissions on the VF-managed Sandy Plains compared to the VF-managed diverse mosaic. Greater biomass in the diverse mosaic pair was primarily due to residual, standing dead forage carried over from the previous year (Figure 1), which may in tandem with increased stock density (4), have prevented selective foraging when steers were concentrated into the smaller VF pastures, particularly in the second rotation into the Salt Flat ecological site, where standing dead vegetation and stock density were greatest. In contrast, lower amounts of residual, standing dead biomass in the Sandy Plains pair of pastures may have facilitated selective foraging, even when the steers were concentrated into the VF sub-pastures. We note that the VF-managed sub-pastures in the Sandy Plains supported lower stock density than their diverse mosaic counterpart, which likely also played a role in mediating behavior-performance-emissions relationships that cannot be examined with our current experimental design.

Another important consideration in spatial distribution management is ensuring cattle have access to water sources (78). In the diverse mosaic block, targeted grazing of the eastern two-thirds of the pasture during the first two rotation intervals required including a long lane along the northern edge of the pasture for cattle to reach the water source. This grazing area polygon placement appears to have affected movement patterns, as these two rotation intervals had a larger difference in distance traveled per day and in area explored per day compared to the controls than the third interval (with no lane necessary) and all intervals in the Sandy Plains pasture (no lane necessary). Visual inspection of animal distribution based on GPS location data showed intensive use of the lane during the first two rotation intervals. This may have resulted in more uneven use of available forage. However, the first interval yielded a decrease and the second an increase in CH_4_ emissions with VF management relative to controls. Further research is needed on how the spatial configuration of virtual sub-pastures, in relation to water and forage conditions, influences cattle distribution, performance, and welfare.

Periodic drought has been shown to decrease species richness, aboveground plant biomass, and concomitant yearling steer performance in grasslands (53, 79–81). This was also true in the current study, where precipitation and ADG were 78 and 82% of the historic average (53, 82). Under conditions of more rapid plant growth and an increase in forb plant components (77, 83), differences in animal performance across VF treatments might be less pronounced, and differences in CH_4_ emissions more pronounced. Furthermore, the data presented here align with outcomes from a recent study conducted in a drought year (i.e., 33% of historical site average; 50), where AHCS routine use was 46% vs. our 48% in the current study and slightly lower CH_4_ emissions, ~200 g CH_4_/hd/d vs. ~214 g/hd/d reported here. Nonetheless, this study offers insights into the spatial behavior and performance of grazing animals, as well as the resulting enteric CH_4_ emissions, during dry conditions typical of the western US shortgrass steppe region. These conditions are becoming increasingly common in a consistent, low productivity megadrought scenario in southwestern US rangelands (84). Future research on grazing animal phenotypic expression in extensive production systems merits further investigation, especially under more conducive non-drought grazing conditions that allow for adequate forage production.

Access to cool-season grass may not have been as critical to maintaining steer growth in the diverse mosaic pastures, but it may remain key for reducing enteric CH_4_ emissions under VF management, as cool-season grasses have less complex cell structures and sugars. Greater CH_4_ production per kg gained by VF-managed steers compared to control steers indicates that increased stocking density in the sub-pastures likely reduced the ability of steers to forage selectively (19). Therefore, a crucial aspect of target-grazing with VF technology is that it requires high capacity in adaptive decision-making to ensure that increased stocking density does not negatively impact selectivity and foraging patterns (4). This element of adjusting VF grazing area for further access to forage also limited capacity for selective foraging in the dry grazing season. Knowing which plant communities produce more enteric emissions at certain times of the year and avoiding grazing them during those periods can potentially reduce the operational footprint of the enterprise. The growing array of predictive grazing tools (85), including Rangeland Analysis Platform (RAP), GrassCast, and the U. S. Drought Monitor, combined with ground-based assessments of animal utilization and knowledge of plant community-specific enteric emissions characteristics, such as methanogenic potential of C_3_- vs. C_4_-photosynthetic pathway forage (86), can assist ranchers in making adaptive decisions that balance animal weight gains with CH_4_ production.

Experimentation using precision livestock management technology, as in the current study, where VF technology, enteric emissions measurement infrastructure, and remote sensing allowed an improved understanding of forage-animal growth performance-emissions relationships, underlies the development of innovative grazing management practices. For instance, a pasture-level evaluation of animal growth performance and AHCS-measured enteric CH_4_ emissions by Thompson, Maciel (87) in two standard Midwest grazing forage mixtures: simple alfalfa:orchardgrass (*Dactylis glomerata L.*) mixture and a complex multi-forage species mixture showed that adopting the complex mixture would not result in improved animal performance or reduced CH_4_ emission intensity compared with a simple mixture for yearling steers and heifers. With this knowledge, a manager could then expect little utility of targeted-grazing management in these plant communities to impact performance and emissions.

Clear trade-offs are associated with employing VF for targeted grazing in extensive semiarid rangeland systems. Manipulating animal distribution into smaller sub-pastures increases stock density, which impacts foraging behavior, diet quality, and animal gains (4, 19, 88). Ranchers will need to employ experiential knowledge to provide sufficient amounts of high-quality forage in each sub-pasture to ensure growth performance and potentially reduce CH_4_ emissions. This management acumen for ranchers is contingent upon knowledge of ecological sites, available plant diversity and phenological growth patterns, and forage quality patterns throughout the grazing season (23, 89). Other benefits of this approach in extensive rangelands include faster-growing livestock and flexible timing of the grazing season terminus, which can increase economic returns (90, 91) and potentially reduce herd-level CH_4_ emissions intensity during their time on rangeland.

The costs of VF implementation and its advantage relative to conventional fencing will vary depending on both the specific application or technology adopted, as well as factors such as topography, cellular coverage, and pasture size (20). Furthermore, as noted in this study, weather impacts on forage quantity and quality, which affect livestock performance related to spatial management of the herd through VF, can have an impact on overall management costs, especially in terms of foregone revenue associated with reduced animal gains. Programs designed to incentivize VF adoption for the purpose of reducing enteric CH_4_ emissions in grazing livestock should address these practical tradeoffs.

## 5 Conclusion

We executed an experiment evaluating the efficacy of VF in implementing rotational grazing management during the growing season in semiarid shortgrass rangeland and quantified the consequences for both cattle weight gain and CH_4_ production. Despite the finding that 16% of the steers in one of the experimental pastures made daily excursions outside the desired grazing areas defined by the virtual fence boundary, the system was still effective in focusing 94–99% of the herd’s locations into desired grazing areas. Notably, VF management did not improve animal performance. Rather, we found consistently lower steer weight gains with VF management across both pairs of experimental pastures. It remains unclear whether this effect arose as a result of VF management on the degree of selective foraging by the cattle, increased energy expenditure, the specific types of plant communities onto which we chose to focus grazing, or some combination of all three factors. Across pastures, overall enteric CH_4_ emissions (mean ± SE) was 214 g CH4/hd/d ± 9 and averaged among VF treatment levels VF-managed steers emitted 216 g CH_4_/hd/d ± 5 vs. 221 g CH_4_/hd/d ± 6 for non-VF-managed steers. Additionally, we found that AHCS subsets of individuals are fair representations of their broader herd in relation to growth performance and spatial behavior. Finally, our results provide no consistent evidence that VF management will simplistically reduce CH_4_ emissions. In the diverse mosaic pair, VF management reduced emissions during the first rotation interval, when VF steers were concentrated on the C_3_-dominated plant community, but increased emissions in the second and third interval when VF steers were concentrated on C_4_-dominated areas. Additionally, due to the spatial arrangement of VF polygons and access to water, we could not fully compare responses to our control animals, which further demonstrates the difficulty of examining forage resource-growth performance-enteric emissions relationship in working livestock production landscapes. In the Sandy Plains pasture pair, VF management did reduce CH_4_ emissions. Yet, the effect of VF management on steer weight gain was larger than the effect on enteric CH_4_ emissions; therefore, VF management did not reduce CH_4_ emissions per kg of beef produced. Although it has been postulated that VF is a potential tool to reduce CH_4_ emissions, our findings show this is not a straightforward outcome, and that greater attention needs to be given to the effect of VF on cattle weight gain and the mechanisms underlying this outcome.

When VF management focused on grazing cool-season grass-dominated areas in the Sandy plains ecological site-associated pasture, enteric CH_4_ emissions were reduced, but animal performance declined by 12% compared to non-VF-managed steers. Conversely, in the diverse mosaic-associated pastures, VF management did not impact growth performance but did increase CH_4_ emission intensity. When VF-managed steers grazed in the warm-season grass-dominant sub-pastures, they likely did not have sufficient access to cool-season grasses to reduce CH_4_ synthesis. Drought-induced reductions in forage quantity and quality impacted the effectiveness of targeted grazing sub-pastures to enhance animal performance while concurrently decreasing enteric CH_4_ emissions. To improve precision grazing strategies, further research should incorporate measures of diet quality to understand the mechanisms driving relationships between animal growth performance and enteric CH_4_ emissions across varying weather and climatic conditions.

## Data Availability

The raw data supporting the conclusions of this article will be made available by the authors, without undue reservation.
